# Exome-Wide Association Study of Competitive Performance in Elite Athletes

**DOI:** 10.3390/genes14030660

**Published:** 2023-03-06

**Authors:** Celal Bulgay, Anıl Kasakolu, Hasan Hüseyin Kazan, Raluca Mijaica, Erdal Zorba, Onur Akman, Isık Bayraktar, Rıdvan Ekmekci, Seyrani Koncagul, Korkut Ulucan, Ekaterina A. Semenova, Andrey K. Larin, Nikolay A. Kulemin, Edward V. Generozov, Lorand Balint, Georgian Badicu, Ildus I. Ahmetov, Mehmet Ali Ergun

**Affiliations:** 1 Sports Science Faculty, Bingol University, 12000 Bingol, Turkey; 2 Faculty of Agriculture, Ankara University, 06000 Ankara, Turkey; 3 Medical Genetics Department, Faculty of Medicine, Near East University, 1010–1107 Nicosia, Cyprus; 4 DESAM Institute, Near East University, 1010–1107 Nicosia, Cyprus; 5 Department of Physical Education and Special Motricity, Faculty of Physical Education and Mountain Sports, Transilvania University, 500068 Braşov, Romania; 6 Sports Science Faculty, Gazi University, 06560 Ankara, Turkey; 7 Sports Science Faculty, Bayburt University, 69000 Bayburt, Turkey; 8 Sports Science Faculty, Alanya Alaaddin Keykubat University, 07450 Alanya, Turkey; 9 Sports Science Faculty, Pamukkale University, 20160 Denizli, Turkey; 10 Sports Department of Medical Biology and Genetics, Marmara University, 34722 Istanbul, Turkey; 11 Department of Molecular Biology and Genetics, Federal Research and Clinical Center of Physical-Chemical Medicine of Federal Medical Biological Agency, 119435 Moscow, Russia; 12 Research Institute of Physical Culture and Sport, Volga Region State University of Physical Culture, Sport and Tourism, 420138 Kazan, Russia; 13 Laboratory of Genetics of Aging and Longevity, Kazan State Medical University, 420012 Kazan, Russia; 14 Department of Physical Education, Plekhanov Russian University of Economics, 115093 Moscow, Russia; 15 Research Institute for Sport and Exercise Sciences, Liverpool John Moores University, Liverpool L3 5AF, UK; 16 Department of Medical Genetics, Faculty of Medicine, Gazi University, 06560 Ankara, Turkey

**Keywords:** athletic performance, athletics, track and field, athletes, aerobic capacity, muscle hypertrophy, sports genetics, WES, EWAS, GWAS

## Abstract

The aim of the study was to identify genetic variants associated with personal best scores in Turkish track and field athletes and to compare allelic frequencies between sprint/power and endurance athletes and controls using a whole-exome sequencing (WES) approach, followed by replication studies in independent cohorts. The discovery phase involved 60 elite Turkish athletes (31 sprint/power and 29 endurance) and 20 ethnically matched controls. The replication phase involved 1132 individuals (115 elite Russian sprinters, 373 elite Russian endurance athletes (of which 75 athletes were with VO_2max_ measurements), 209 controls, 148 Russian and 287 Finnish individuals with muscle fiber composition and cross-sectional area (CSA) data). None of the single nucleotide polymorphisms (SNPs) reached an exome-wide significance level (*p* < 2.3 × 10^−7^) in genotype–phenotype and case–control studies of Turkish athletes. However, of the 53 nominally (*p* < 0.05) associated SNPs, four functional variants were replicated. The *SIRT1* rs41299232 G allele was significantly over-represented in Turkish (*p* = 0.047) and Russian (*p* = 0.018) endurance athletes compared to sprint/power athletes and was associated with increased VO_2max_ (*p* = 0.037) and a greater proportion of slow-twitch muscle fibers (*p* = 0.035). The *NUP210* rs2280084 A allele was significantly over-represented in Turkish (*p* = 0.044) and Russian (*p* = 0.012) endurance athletes compared to sprint/power athletes. The *TRPM2* rs1785440 G allele was significantly over-represented in Turkish endurance athletes compared to sprint/power athletes (*p* = 0.034) and was associated with increased VO_2max_ (*p* = 0.008). The *AGRN* rs4074992 C allele was significantly over-represented in Turkish sprint/power athletes compared to endurance athletes (*p* = 0.037) and was associated with a greater CSA of fast-twitch muscle fibers (*p* = 0.024). In conclusion, we present the first WES study of athletes showing that this approach can be used to identify novel genetic markers associated with exercise- and sport-related phenotypes.

## 1. Introduction

Whether pure talent or long-term experiences promotes athletic performance is one of the questionable issues [1]. Progression in the sport sciences has underlined that athletic performance was a phenomenon affected by lots of factors including physiology and environment [2]. Recent studies have also figured out the possible association of the genetic background of the athletes in their high personal performances, resulting in the rise of a novel scientific branch, called sport genetics [3,4].

Sport genetics could be defined as the investigation of the genes and their molecular mechanisms affecting athletic performance and the determination of the possible association of the variants, especially single nucleotide polymorphisms (SNPs), with diverse athletic parameters including branch or personal performances [5]. According to the studies on sport genetics, 66% of athletic performance has been linked to the genetic background [6]. Moreover, physical parameters were also associated with the genetic background. For instance, 44–68% of endurance and 49–56% of muscular force were shown to be affected by genetic variations [7,8]. Thus, both genetics and the environment, which would influence each other, have key roles in athletic performance [9,10]. For example, training periods to reach a performance level were proved to be linked to the genetic background of the athletes [9].

Recently, identification of candidate genes and/or variants associated with sports parameters has greatly attracted scientists. Until now, more than 235 genetic variants have been linked to the athletic parameters [10,11]. However, the results of the single-gene and/or variant approach may mislead, owing to the ignorance of the other related genes and/or variants. Consequently, it was realized that the results for the associations of each gene and/or variant were controversial [12]. Hence, multigenetic factors should be targeted to totally explore the possible associations. In parallel, several genome-wide association studies (GWAS) have been conducted on sports genetics. GWAS is a powerful technique to cover all known or unknown SNPs [13,14,15]. GWAS has proposed novel associated genes and/or SNPs for the athletic parameters such as endurance, aerobic capacity, metabolism, and muscle fiber composition [16,17]. However, the complexity and cost of GWAS limit such studies, and pilot experiments are suggested [18]. Exome-wide association studies (EWAS) could be an alternative to overcome the problems with GWAS. EWAS has also been previously chosen as a strategy to find the possible associations in the sports genetics [19].

The aim of the present study was to identify genetic variants associated with personal best scores in Turkish track and field athletes and to compare allelic frequencies between sprint/power and endurance athletes and controls using a whole-exome sequencing approach, followed by replication studies in independent cohorts of athletes and controls.

## 2. Materials and Methods

### 2.1. Ethical Approval

The study was carried out in accordance with the Declaration of Helsinki, and approval was obtained from the Gazi University Non-Interventional Clinical Research Ethics Committee (with the decision dated 5 April 2021 and numbered 09) and from the Ethics Committee of the Federal Research and Clinical Center of Physical-Chemical Medicine of the Federal Medical and Biological Agency of Russia (Approval number 2017/04).

### 2.2. Participants

#### 2.2.1. The Turkish Cohorts

The Turkish study involved 60 elite athletes (sprint/power: 11 females (35.5%) and 20 males (64.5%); endurance: 10 females (34.5%) and 19 males (65.5%); mean age ± SD: 25.1 ± 4.8; height (cm): 174.97 ± 7.9; body weight (kg) 72.5 ± 22.4; sport experience (year) = 9.4 ± 4.8; personal best (PB) = 1005.63 ± 94.55) licensed in different clubs and affiliated with the Turkish Athletics Federation. The number of controls (non-athletes) was 20 (6 females (30.0%) and 14 males (70.0%); mean age ± SD: 23.5 ± 7.1), and they were healthy unrelated citizens of Turkish descent without any competitive sports experience.

The athletes were categorized as either sprint/power or endurance athletes as determined by the distance, duration, and energy requirements of their events. All athletes were nationally ranked in the top ten in their sports discipline and had participated in international competitions such as the Olympic Games, European Championships, Universiade, Mediterranean Games, and Balkan Championship. The sprint/power group included sprint and power athletes whose events demand predominantly anaerobic energy production. The athletes in this group (*n* = 31) were 100–400 m runners (*n* = 9), jumpers (*n* = 3), and throwers (*n* = 19). The endurance athlete group (*n* = 29) included athletes competing in long-distance events demanding predominantly aerobic energy production. This group included 3000 m (*n* = 12), 5000 m (*n* = 5), 10,000 m (*n* = 4), and marathon (*n* = 8) runners. The informed voluntary consent and demographic information forms were obtained from the participants before the measurements. The International Association of Athletics Federations (IAAF) score scale was used to determine the performance levels of the athletes, depending on their personal best/competitive performance [20]. For instance, the IAAF score scale of a male athlete who runs 100 m in 10.05 sec is 1189, while that of a marathon runner who completes the race in 2 h 20 min 11 sec is 997. Thus, the performance scale of the marathon runner is less than that of the 100 m runner. The IAAF scales are useful for the determination of performances of athletes from diverse athletics events and genders.

#### 2.2.2. The Russian Cohorts

The Russian case–control study involved 488 elite athletes (293 males and 195 females), of whom 115 were elite sprint/power athletes (29 100–400 m runners, 38 500–1000 m speed skaters, 22 sprint cyclists, 26 50 m swimmers), and 373 were elite endurance athletes (52 rowers, 32 biathletes, 7 long-distance cyclists, 30 kayakers and canoers, 37 middle- and long-distance speed skaters, 92 cross-country skiers, 63 middle- and long-distance runners, 31 middle- and long-distance swimmers, 8 race walkers, and 21 triathletes). The athletes were Russian national team members (participants and prize winners in international competitions) who had never tested positive for doping. Of 373 endurance athletes, 46 male endurance athletes (rowers, kayakers, speed skaters, biathletes, and cross-country skiers) and 29 female endurance athletes (rowers, kayakers, speed skaters, biathletes, and cross-country skiers) participated in the study of aerobic performance. Controls were 209 healthy and unrelated citizens of Russia without any competitive sport experience.

The Russian muscle biopsy study involved 148 physically active participants of Russian origin (99 males: mean age ± SD: 30.4 ± 7.9 years; 49 females: mean age ± SD: 27.1 ± 7.3 years).

#### 2.2.3. The Finnish Cohort

The Finnish muscle biopsy study (replication phase) involved 287 individuals (167 males, age 59.5 ± 8.1 years; 120 females, age 60.7 ± 7.4 years) from the FUSION study as previously described [21].

### 2.3. Evaluation of Muscle Fiber Composition by Immunohistochemistry

#### 2.3.1. Russian Study

Vastus lateralis samples were obtained from the left legs of the participants using the modified Bergström needle procedure with aspiration under local anesthesia using 2% lidocaine solution. Serial cross-sections (7 μm) were obtained from frozen samples. The sections were then incubated at RT in primary antibodies against slow or fast isoforms of the myosin heavy chains, as previously described [17,22].

#### 2.3.2. Finnish Study

Muscle fiber composition in 287 Finnish individuals was estimated based on the expression of the myosin heavy chain 1 (MYH1), myosin heavy chain 2 (MYH2), myosin heavy chain 7 (MYH7), Ca^2+^ ATPase A1, and Ca^2+^ ATPase A2 genes, as previously described [21]. 

### 2.4. VO_2max_ Measurement

Maximal oxygen consumption rate (VO_2max_) in rowers, kayakers, speed skaters and biathletes was determined using an incremental test to exhaustion on specific ergometers. VO_2max_ was determined breath-by-breath using a MetaLyzer II (Cortex Bio-physik, Leipzig, Germany), MetaMax 3B (Cortex Biophysik, Leipzig, Germany) or MetaMax 3B-R2 gas analysis systems (Cortex Biophysik, Leipzig, Germany), as previously described [23]. 

### 2.5. Whole-Exome Sequencing (WES)

The peripheral blood obtained from the participants was processed to isolate total DNA by DNeasy Blood and Tissue Kit (Qiagen, Hilden, Germany) according to the manufacturer’s instructions. Next, qualities of isolated DNA were checked by 1% agarose gel, and the concentrations were determined by a NanoDrop (NanoDrop 1000 Spectrophotometer V3.8; Thermo Scientific, Waltham, MA, USA). WES was performed after library preparation by the Twist Human Comprehensive Exome Panel (Twist Biosciences, San Francisco, CA, USA) according to the supplier’s instructions. Briefly, enzymatic DNA fragmentation was performed, and Twist Hybridization probes and Dynabeads™ MyOne™ Streptavidin T1 (Invitrogen, Carlsbad, CA, USA) were used for the hybridization. After the steps of library enrichment and determination of the library sizes, the samples were uploaded to the flow cells and the run was performed by Illumina NextSeq500 (Illumina Inc., San Diego, CA, USA). Average read depth was aimed as minimum 200×. Raw data were processed to by the Genome Analysis Toolkit (GATK)’s [24]. The HaplotypeCaller program was used to obtain Binary Alignment Map (BAM) files and subsequently produce an output Variant Call Format (VCF) file via the GRCh38/hg38 reference genome. Finally, variants were annotated by ANNOVAR [25].

### 2.6. Data Extraction

As the primary evaluation of the data, the VCF files were combined, and 511,061 variants were detected. Only SNPs were analyzed in the context of the present study. The variants with a minor allele frequency (MAF) < 0.01, incorrectly annotated, and non-autosomal were eliminated, and 219,232 SNPs were further evaluated.

### 2.7. Genotyping

DNA samples from Russian individuals were obtained from leukocytes (venous blood). DNA extraction and purification from blood samples were performed using commercial kits (Techno-sorb), according to the manufacturer’s instructions (Techno-clon, Moscow, Russia). Genotyping of the candidate SNPs from the discovery phase was performed using microarray technology [26]. 

DNA samples from Finnish individuals were extracted from the blood, and the polymorphisms were genotyped using the HumanOmni2.5–4v1_H BeadChip array (Illumina, San Diego, CA, USA), as previously described [21].

### 2.8. Statistical Analyses

Association analyses of Turkish data were performed by a Chi-square test using thet R program [27]. During the EWAS, the unified mixed-model method [27] was used.

y = Xβ + Sτ + e(1)

where y is the phenotypic observation; Xβ is the fixed effect; and Sτ is the SNP effect [27]. The statistical significance probabilities of the SNP effects were converted to −log10p. The results of EWAS analyses were presented as a Manhattan Plot. The exome-wide significance level was set at *p* < 2.3 × 10^−7^ (i.e., 0.05/219,232 SNPs).

Statistical analyses of Russian and Finnish data were conducted using GraphPad InStat Version 3.05 (GraphPad Software, Inc., San Diego, CA, USA) software. The PLINK 1.9 program (National Institutes of Health, Bethesda, MD, USA) was used to perform genetic data quality control, and PLINK 2.0 was used to perform principal component analysis and association testing via generalized linear models. Bcftools was used for vcf file conversion. The phasing and imputation of genotypes were completed using the shapeit2 and impute2 programs. Differences in phenotypes between groups were analyzed using regression analysis adjusted for covariates. The chi-square test (χ^2^) was used to test for the presence of the Hardy–Weinberg equilibrium (HWE). Thereafter, the frequencies of genotypes or alleles were compared between sprint/power and endurance athletes and controls using Fisher’s exact test. All data are presented as means (SD). The *p*-values < 0.05 were considered statistically significant.

## 3. Results

### 3.1. Discovery Phase

**Figure 1:**
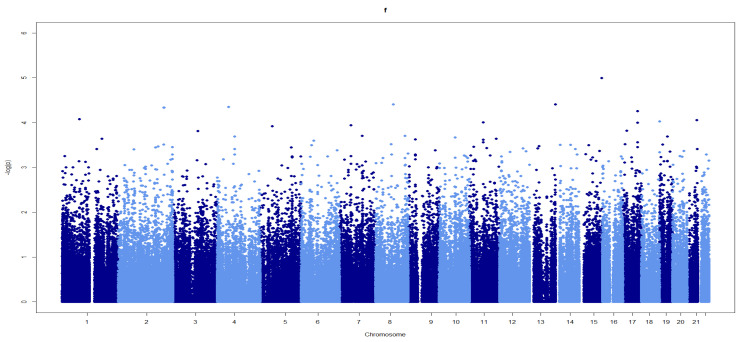
Manhattan plot showing associations between SNPs across chromosomes and personal bests in elite Turkish athletes. Light and dark blue: the illustration separating the consecutive chromosomes for comprehensibility.

None of the SNPs reached an exome-wide significance level (*p* < 2.3 × 10^−7^) in genotype–phenotype and case–control studies of Turkish athletes (Figure 1). The only SNP that was close to the threshold (*p* = 1.0 × 10^−5^) was rs8037843 in the Pyroglutamyl-Peptidase I Like (*PGPEP1L*) gene (Figure 1). Although rs8037843 correlated with personal bests in athletes, there were no allelic differences between the Turkish and Russian endurance and sprint/power athletes and controls with respect to this SNP (*p* > 0.05).

**Figure 2:**
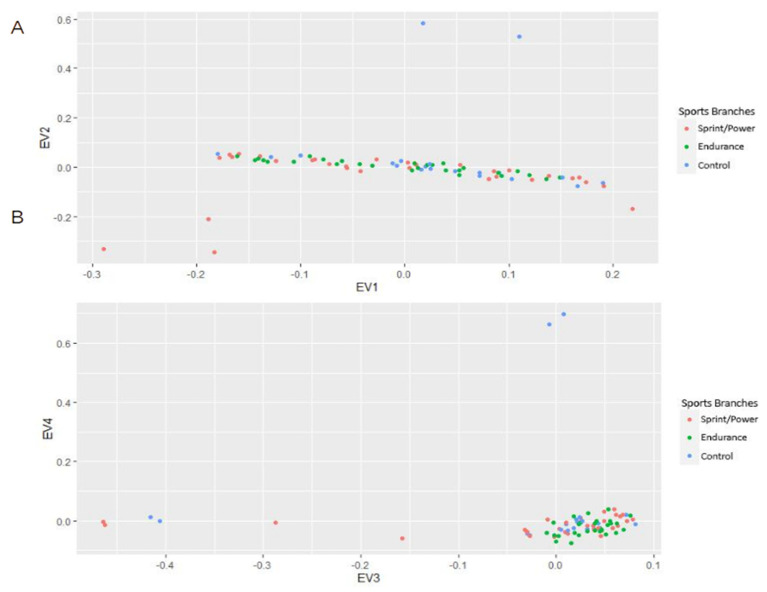
Principal component analysis on the SNP matrix showing genotype distributions across different groups in EV1−EV2 (**A**) and EV3−EV4 (**B**) planes.

The genotypic differences between the groups were evaluated by principal component analysis on an SNP matrix (PCA). PCA of the genotyping data pointed out no significant influence of sport disciplines (Figure 2) on genotype distributions.

**Figure 3:**
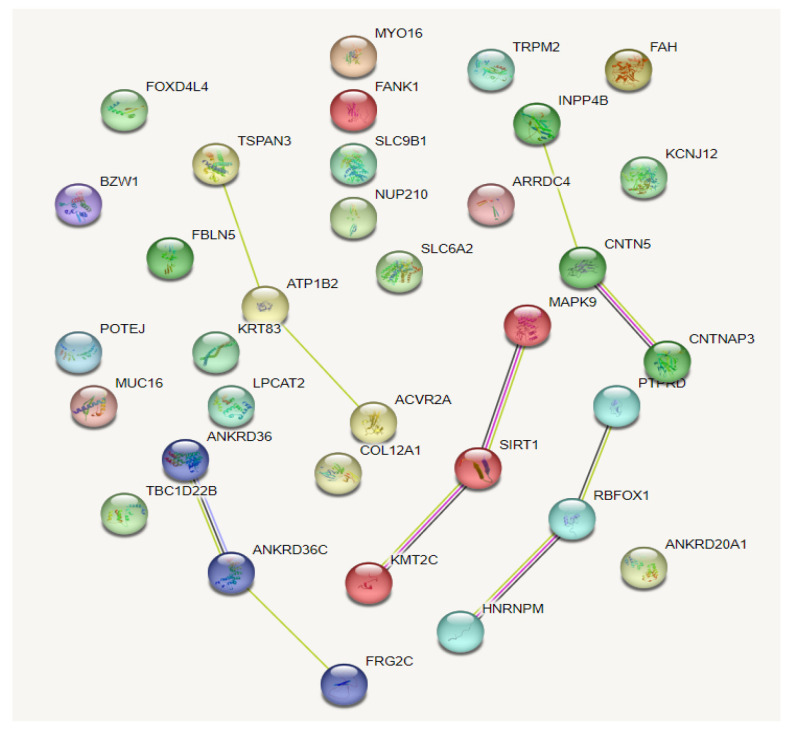
Interaction of and clustering of the protein-coding genes on which deviated frequencies of the SNPs were realized between the sprint/power and endurance groups.

Comparisons of allelic frequencies between three groups (endurance vs. sprint/power athletes; endurance athletes vs. controls; sprint/power athletes vs. controls) showed 53 SNPs whose frequencies were significantly differentiated between the sprint/power and endurance group (but not in the separate sub-groups of female and male athletes due to low sample sizes) (Supplementary Table S1). The genes in which these SNPs were located were further analyzed by the String database (v.11.5; https://string-db.org/, accessed on 10 December 2022) for the functional interaction and pathway analyses. The results showed minimal interactions between the proteins, and the Markov Cluster Algorithm (MCL) option in the database demonstrated five clusters (Figure 3).

### 3.2. Replication Studies

Of the 53 nominally (*p* < 0.05) associated SNPs, four variants were replicated in the following studies involving Russian and Finnish individuals. More specifically, the *SIRT1* rs41299232 G allele was significantly over-represented in Turkish (44.0 vs. 4.0%; *p* = 0.047) and Russian (63.5 vs. 55.4%; *p* = 0.018) endurance athletes compared to sprint/power ones and was associated with increased relative VO_2max_ (C/C (*n* = 9): 63.2 (8.0) mL/min/kg, C/G (*n* = 35): 64.3 (7.0) mL/min/kg, G/G (*n* = 31): 66.2 (6.4) mL/min/kg; *p* = 0.037 adjusted for sex), and a greater proportion of slow-twitch muscle fibers in Finnish subjects (C/C (*n* = 45): 42.8 (12.7)%, C/G (*n* = 147): 44.4 (16.0)%, G/G (*n* = 95): 47.0 (13.7)%; *p* = 0.035 adjusted for age and sex).

The *NUP210* rs2280084 A allele was significantly predominant in Turkish (68.0 vs. 34.0%, *p* = 0.044) and Russian (59.2 vs. 50.0%, *p* = 0.012) endurance athletes compared to the sprint/power group. In addition, the rs2280084 A allele was over-represented in highly elite Russian endurance athletes (*n* = 119) compared to controls (64.1 vs. 52.9%, *p* = 0.003).

The frequency of the *TRPM2* rs1785440 G allele was significantly higher in Turkish endurance athletes compared to sprint/power athletes (57.0 vs. 18.0%, *p* = 0.034) and was associated with increased VO_2max_ (A/A (*n* = 1): 58.3 (0) mL/min/kg, A/G (*n* = 16): 63.2 (6.9) mL/min/kg, G/G (*n* = 58): 65.6 (6.8) mL/min/kg; *p* = 0.008).

The *AGRN* rs4074992 C allele was significantly over-represented in Turkish sprint/power athletes compared to endurance athletes (83.0 vs. 44.0%, *p* = 0.037) and was associated with a greater CSA of fast-twitch muscle fibers in physically active Russian individuals (*p* = 0.024 adjusted for sex, age, type, and level of physical activity).

## 4. Discussion

Athletic performance and branches have widely been proved to be a result of the combination of environmental and genetic factors [1,28]. The latter, named as sports genetics, has attracted sports scientists since it was relatively a new branch [10]. The studies on sport genetics have focused on single-gene and/or SNP alteration between the sport branches, which may mislead [12]. Hence, studies aiming at the involvement of multigenetic factors are needed. Limited studies, but not on the Turkish population, have reported GWAS results in sports genetics [10,13,14,16,29]. Thus, the present study focused on the assessment of the multigenetic factors in the elite sprint/power and endurance athletes using the WES approach. Although WES is not a cumulative approach compared to whole-genome sequencing (WGS), it may be advantageous for a pilot study such as the presented one to eliminate the analysis efforts and cost problems.

In our present study, we could not detect any SNPs whose frequencies reached an exome-wide significance. The primary problem with such studies would be the limitations with the number of participants [28]. Sport genetics has been established on a population- and sport-branch-specific manner. However, the restricted number of elite athletes would be a challenge to conclude exact findings [5]. Still, by the fact that the number of participants in such studies could affect the results according to the literature [30], such studies are still needed as a pilot comprehensive report to guide both the geneticists and sport scientists.

By the lack of associations with a threshold of *p* < 2.3 × 10^−7^, we further compared the frequencies of the SNPs between the sprint/power and endurance groups with *p* < 0.05 using the Chi square test. The results pointed out 53 SNPs whose frequencies significantly differentiated between the sport groups (*p* < 0.05; Supplementary Table S1). Of the 53 SNPs, four functional (i.e., affecting gene expression) variants located on the (or near) *SIRT1*, *NUP210*, *TRPM2*, and *AGRN* genes were replicated in Russian and Finnish individuals with consistent effects.

The *SIRT1* gene encodes the sirtuin 1 protein which is considered as a functional regulator (through the deacetylation and activation) of peroxisome proliferator-activated receptor-γ coactivator (PGC-1α) that induces a metabolic gene transcription program of mitochondrial fatty acid oxidation (one of the positive factors of aerobic capacity) [31]. In our study, we found that the *SIRT1* rs41299232 G allele was significantly over-represented in Turkish and Russian endurance athletes compared to sprint/power ones and was associated with increased VO_2max_ and a greater proportion of slow-twitch muscle fibers. Both phenotypes are considered advantageous for endurance athletes. According to the GTEx portal [32], the *SIRT1* rs41299232 (intronic variant) is significantly (*p* = 4.2 × 10^−33^) associated with the altered expression of the *SIRT1* gene in the whole blood. Previously, the rs41299232 G allele was reported to be associated with an increased red blood cell count (*p* = 0.0000015), higher hemoglobin concentration (*p* = 0.0032), and higher physical activity (*p* = 0.0031) in the UK Biobank cohort [33], which is in line with our findings.

The *NUP210* gene encodes nucleoporin 210 (a membrane-spanning glycoprotein), which is a major component of the nuclear pore complex. Previously, the *NUP210* has been shown as a critical regulator of muscular and neuronal differentiation [34]. Muscle function experiments in mice have shown that *Nup210* is required for muscle endurance during voluntary running and muscle repair after injury [35]. In our study, we found that the frequency of the *NUP210* rs2280084 A allele was significantly higher in Turkish and Russian endurance athletes compared to sprint/power athletes, as well as in highly elite Russian endurance athletes compared to controls. According to the GTEx portal [32], the *NUP210* rs2280084 (missense variant) is significantly associated with changed expression of the *NUP210* gene in the brain (*p* = 3.9 × 10^−11^) and the whole blood (*p* = 0.000032).

The *TRPM2* gene encodes the transient receptor potential cation channel subfamily M member 2 protein. *TRPM2* plays an important role in a variety of cellular functions, including cell proliferation, insulin release, cell motility, and cell death [36,37]. Recently, it has been shown that TRPM2-mediated Ca^2+^ signaling is required for training-induced improvement in skeletal muscle mitochondrial functions and fiber-type transition in mice [38]. In our study, we found that the *TRPM2* rs1785440 G allele was significantly over-represented in Turkish endurance athletes compared to sprint/power ones and was associated with increased VO_2max_ in Russian athletes. According to the GTEx portal [32], the *TRPM2* rs1785440 (intronic variant) is significantly (*p* = 2.5 × 10^−12^) associated with an altered expression of the *TRPM2* gene in the skeletal muscle.

The *AGRN* gene encodes the agrin protein, which regulates the maintenance of the neuromuscular junction [39]. Previous studies have linked the *AGRN* gene variants with sarcopenia-related traits (muscle mass and strength) and congenital myasthenia [39,40]. Furthermore, *Agrn* gene expression has been shown to be upregulated after progressive weighted wheel running in mice [41]. In our study, we found that the *AGRN* rs4074992 C allele was significantly over-represented in Turkish sprint/power athletes compared to endurance athletes and was associated with a greater CSA of fast-twitch muscle fibers in physically active Russian individuals. Muscle fiber size is a surrogate indicator of muscle mass and is positively associated with power and strength [42,43,44]. According to the GTEx portal [32], the *AGRN* rs4074992 (intergenic variant) is significantly (*p* ≥ 3.8 × 10^−8^) associated with altered expression of the *AGRN* gene in multiple tissues. Previously, the rs4074992 C allele has been reported to be associated with increased appendicular lean mass (*p* = 0.0031) in the UK Biobank cohort [33], which is in line with our findings.

Like the present study, a study in diverse populations conducted in the literature reported that none of the SNPs reached genome-wide significance with the endurance athlete status [14]. Nonetheless, others reported the associations of the specific SNPs with different exercise-related parameters [15,16,17,45,46,47,48]. However, the number of participants in those studies was increased by the involvement of the athletes from close countries. Importantly, only one study was able to present a clear association between Tatar wrestlers and a specific SNP in an athletic group with limited participants [5]. Therefore, we can also underline that such studies are critically influenced by the populations, number of the participants, and sport branches.

The present study had some limitations that may be common in other sport genetics studies. These limitations could be the restricted number of participants in the discovery phase (*n* = 80), heterogeneity in the branches, diverse ethnicity in the Turkish population, lack of controllability of environmental factors, and ignorance of the epigenetic mechanisms. On the other hand, we regard that we were able to reduce the probability of obtaining false-positive results by replicating our initial findings—a widely used approach in sports genetics—in the larger cohorts of Russian (*n* = 845) and Finnish individuals (*n* = 287) [14,23,49]. Still, the present study figured out four important SNPs that would further be analyzed in the next studies.

## 5. Conclusions

In conclusion, by conducting the first comprehensive WES study on elite athletes, we showed that the *SIRT1* rs41299232 G, *NUP210* rs2280084 A, and *TRPM2* rs1785440 G alleles are associated with endurance athlete status, whereas the *AGRN* rs4074992 C allele is linked with sprint/power athlete status and muscle fiber hypertrophy. Our data indicate that the WES approach followed by replication studies can be used to identify novel genetic markers associated with exercise- and sport-related phenotypes.

## Data Availability

The data presented in this study are available on request from the corresponding author.

## Supplementary Materials

The following supporting information can be downloaded at: https://www.mdpi.com/article/10.3390/genes14030660/s1, Table S1: SNPs were significantly associated with athlete status in the Turkish cohorts of endurance athletes, sprint/power athletes, and controls.
