# Supplementary material for: Exome-Wide Association Study of Competitive Performance in Elite Athletes
Source: Genes (Basel). 2023 Mar 6;14(3):660. doi: 10.3390/genes14030660 (PMC10048216; doi:10.3390/genes14030660)
Supplement: Supplementary file 1 [file genes-14-00660-s001.zip › genes-2250845-supplementary.pdf]

Supplementary Table S1. SNPs associated with athlete status in the Turkish cohorts of endurance athletes, power athletes and controls.

Gene	Locus (CRCh38.p14)	Polymorphism	REF allele	ALT allele	Endurance vs sprint/power *p* value	Endurance vs controls *p* value	Sprint/power vs controls *p* value	Allelic frequencies in sprint/power athletes	Allelic frequencies in controls	Allelic frequencies in endurance athletes
ACVR2A	chr2_147926350	rs12620026	A	T	0.038	0.048	0.919	A (38%)<br>T (62%)	A (62%)<br>T (38%)	A (84%)<br>T (16%)
ANKRD20A1	chr9_67901030	rs62542472	C	T	0.017	0.488	0.002	C (98%)<br>T (2%)	C (55%)<br>T (45%)	C (50%)<br>T (50%)
ANKRD36	chr2_97164236	rs200092139	G	A	0.028	0.285	0.001	G (100%)	G (52%)<br>A (48%)	G (55%)<br>A (45%)
	chr2_97163407	rs62153885	T	G	0.026	0.450	0.139	T (98%)<br>G (2%)	T (100%)	T (53%)<br>G (47%)
	chr2_97164279	rs201424946	G	T	0.023	0.441	0.002	G (100%)	G (58%)<br>T (42%)	G (53%)<br>T (47%)
	chr2_97142481	rs79070930	A	G	0.023	0.498	0.105	A(100%)	A(98%)<br>G(2%)	A (53%)<br>G (47%)
	chr2_97142492	rs74969160	G	A	0.018	0.398	0.123	G (100%)	G (100%)	G (51%)<br>A (49%)
ANKRD36C	chr2_95954026	rs77522461	G	A	0.018	0.371	0.001	G (100%)	G (53%)<br>A (47%)	G (51%)<br>A (49%)
	chr2_95941237	rs79451673	T	C	0.018	0.398	0.123	T (100%)	T (100%)	T (51%)<br>C (49%)
ARRDC4	chr15_97965441	rs8031829	C	A	0.025	0.331	0.195	C (66%)<br>A (34%)	C (65%)<br>A (35%)	C (29%)<br>A (71%)
ATP1B2	chr17_7654516	rs1642764	C	T	0.041	0.512	0.157	C (64%)<br>T (36%)	C (60%)<br>T (40%)	C (31%)<br>T (69%)
BZW1	chr2_200815208	rs12475785	G	T	0.040	0.746	0.081	G (69%)<br>T (31%)	G (57%)<br>T (43%)	G (34%)<br>T (66%)
CNTN5	chr11_99819697	rs7125822	T	G	0.018	0.496	0.002	T (100%)	T (57%)<br>G (43%)	T (51%)<br>G (49%)
CNTNAP3	chr9_39088492	rs1758499	T	A	0.018	0.432	0.002	T (100%)	T (55%)<br>A (45%)	T (51%)<br>A (49%)
COL12A1	chr6_75134129	rs1332778	T	C	0.037	0.286	0.296	T (62%)<br>C (38%)	T (67%)<br>C (33%)	T (29%)<br>C (71%)
FANK1	chr10_125896805	rs5013165	C	T	0.028	0.335	0.002	C (100%)	C (55%)<br>T (45%)	C (55%)<br>T (45%)
	chr10_125896794	rs74162872	G	A	0.028	0.504	0.123	G (100%)	G (100%)	G (55%)<br>A (45%)

FBLN5	chr14_91937316	rs2474028	T	C	0.033	0.746	0.069	T (70%)<br>C (30%)	T (58%)<br>C (42%)	T (34%)<br>C (66%)
FOXD4L4	chr9_65737152	rs1212108435	C	A	0.028	0.504	0.123	C (100%)	C (100%)	C (55%)<br>A (45%)
FRG2C	chr3_75665747	rs62247159	G	A	0.023	0.450	0.123	G (100%)	G (100%)	G (53%)<br>A (47%)
HNRNPM	chr19_8445260	rs11881939	T	C	0.026	0.064	0.705	T (41%)<br>C (59%)	T (75%)<br>C (25%)	T (93%)<br>C (7%)
INPP4B	chr4_142081862	rs1353603	T	C	0.042	0.344	0.268	T (75%)<br>C (25%)	T (83%)<br>C (17%)	T (39%)<br>C (61%)
KMT2C	chr7_152230042	rs62481502	A	G	0.042	0.198	0.450	A (50%)<br>G (50%)	A (100%)	A (100%)
KRT83	chr12_52314457	rs2852456	G	A	0.043	0.326	0.288	G (70%)<br>A (30%)	G (77%)<br>A (23%)	G (36%)<br>A (64%)
AGRN	chr1_1067673	rs4074992	C	T	0.037	0.847	0.057	C (83%)<br>T (17%)	C (70%)<br>T (30%)	C (44%)<br>T (56%)
MAPK9	chr5_180238572	rs4700729	A	G	0.039	0.710	0.089	A (85%)<br>G (15%)	A (78%)<br>G (22%)	A (46%)<br>G (54%)
MUC3A& LOC105375431	chr7_100957146	rs1378797034	G	A	0.028	0.285	0.001	G (100%)	G (52%)<br>A (48%)	G (55%)<br>A (45%)
	chr7_100957183	rs1420864111	C	A	0.028	0.285	0.001	C (100%)	C (53%)<br>A (47%)	C (55%)<br>A (45%)
	chr7_100959537	rs74318947	A	T	0.023	0.326	0.001	A (100%)	A (53%)<br>T (47%)	A (53%)<br>T (47%)
	chr7_100960407	rs73163759	T	C	0.023	0.326	0.001	T (100%)	T (53%)<br>C (47%)	T (53%)<br>C (47%)
	chr7_100957015	rs1194458035	T	C	0.018	0.372	0.001	T (100%)	T (52%)<br>C (48%)	T (51%)<br>C (49%)
	chr7_100959517	rs77153975	A	G	0.018	0.372	0.001	A (100%)	A (53%)<br>G (47%)	A (51%)<br>G (49%)
	chr7_100957158	rs1378797034	T	C	0.043	0.256	0.002	T (100%)	T (55%)<br>C (45%)	T (58%)<br>C (42%)
	chr7_100956464	rs1373570695	T	G	0.018	0.398	0.123	T (100%)	T(100%)	T (51%)<br>G (49%)
	chr7_100956873	rs1024883687	A	G	0.018	0.398	0.123	A (100%)	A (100%)	A (51%)<br>G (49%)
	chr7_100957083	rs1245494471	C	T	0.018	0.398	0.123	C (100%)	C (100%)	C (51%)<br>T (49%)
	chr7_100959437	rs78538898	T	C	0.018	0.398	0.123	T (100%)	T (100%)	T (51%)<br>C (49%)
	chr7_100959563	rs73714242	C	T	0.023	0.450	0.123	C (100%)	C (100%)	C (53%)

										T (47%)
	chr7_100957117	rs1223084315	A	G	0.043	0.621	0.123	A (100%)	A (100%)	A (58%)<br>G (42%)
MUC16	chr19_8851096	rs2972591	A	G	0.030	0.390	0.181	A (79%)<br>G (21%)	A (80%)<br>G (20%)	A (39%)<br>G (61%)
MYO16	chr13_109124983	rs157023	G	T	0.039	1.000	0.039	G (74%)<br>T (26%)	G (55%)<br>T (45%)	G (37%)<br>T (63%)
NOX5& SPESP1	chr15_68946106	rs3743093	G	A	0.028	1.000	0.028	G (72%)<br>A (28%)	G (50%)<br>A (50%)	G (34%)<br>A (66%)
NUP210	chr3_13317933	rs354477	G	A	0.038	1.000	0.038	G (79%)<br>A (21%)	G (60%)<br>A (40%)	G (41%)<br>A (59%)
	chr3_13354079	rs2280084	C	A	0.044	0.909	0.057	C (66%)<br>A (34%)	C (50%)<br>A (50%)	C (32%)<br>A (68%)
POTEJ	chr2_130656603	rs62165276	C	T	0.031	0.382	0.003	C (96%)<br>T (4%)	C (55%)<br>T (45%)	C (53%)<br>T (47%)
PTPRD	chr9_8341445	rs3736381	T	C	0.046	0.613	0.013	T (83%)<br>C (17%)	T (55%)<br>C (45%)	T (46%)<br>C (54%)
RBFOX1	chr16_5239841	rs1279779604	G	T	0.023	0.282	0.002	G (100%)	G (55%)<br>T (45%)	G (53%)<br>T (47%)
SIRT1	chr10_67887701	rs41299232	C	G	0.047	0.798	0.083	C (96%)<br>G (4%)	C (90%)<br>G (10%)	C (56%)<br>G (44%)
SLC6A2	chr16_55684972	rs2270935	T	C	0.043	0.473	0.185	T (33%)<br>C (67%)	T (88%)<br>C (12%)	T (75%)<br>C (25%)
SLC9B1	chr4_102901335	rs3974500	G	A	0.023	0.450	0.123	G (100%)	G (100%)	G (53%)<br>A (47%)
TBC1D22B	chr6_37314058	rs149705	T	C	0.043	0.761	0.021	T (82%)<br>C (18%)	T (57%)<br>C (43%)	T (44%)<br>C (56%)
TRPM2	chr21_44406920	rs1785440	A	G	0.034	0.398	0.009	A (82%)<br>G (18%)	A (50%)<br>G (50%)	A (43%)<br>G (57%)
TSPAN3	chr15_77071109	rs11072641	T	C	0.013	0.429	0.086	T (98%)<br>C (2%)	T (92%)<br>C (48%)	T (48%)<br>C (52%)
